# Supplementary figures and images for: Human DICER helicase domain recruits PKR and modulates its antiviral activity
Source: PLoS Pathog. 2021 May 13;17(5):e1009549. doi: 10.1371/journal.ppat.1009549 (PMC8118307; doi:10.1371/journal.ppat.1009549)

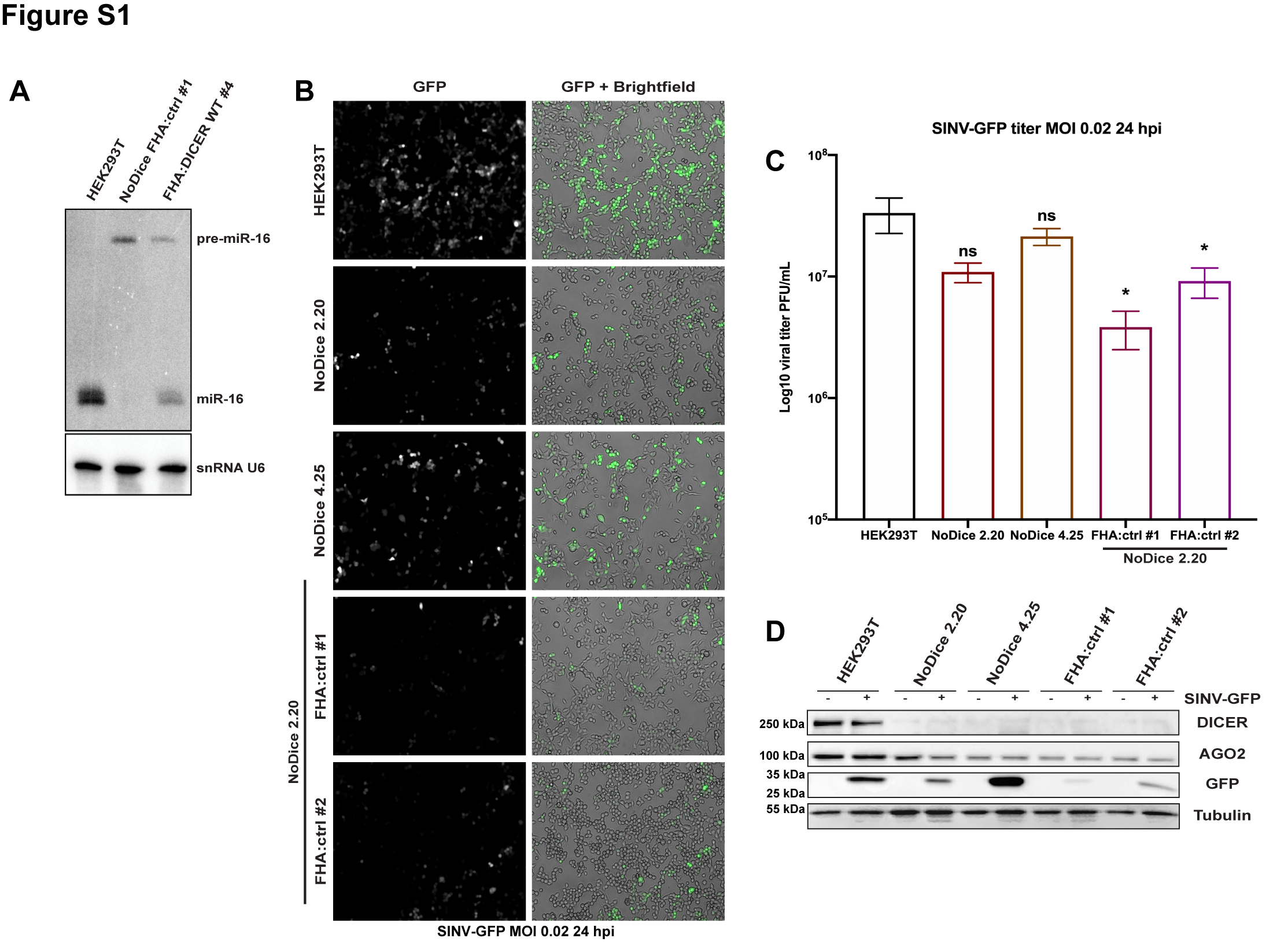

Supplement: S1 Fig — A. miR-16 expression analyzed by northern blot in HEK293T, NoDice FHA:ctrl #1 and FHA:DICER WT #4 cell lines. Expression of snRNA U6 was used as loading control. B. Representative GFP pictures of HEK293T, NoDice 2.20, NoDice 4.25, NoDice FHA:ctrl #1 and NoDice FHA:ctrl #2 cells infected with SINV-GFP at an MOI of 0.02 for 24 h. The left panel corresponds to GFP signal and the right panel to a merge of GFP signal and the corresponding brightfield. Pictures were taken with a 5x magnification. hpi: hours post-infection. C. Mean (+/- SEM) of SINV-GFP viral titers in cells infected at an MOI of 0.02 for 24 h (n = 3) from plaque assay quantification. * p < 0.05, ns: non-significant, ordinary one-way ANOVA test with Bonferroni correction. D. Western blot analysis of DICER, AGO2 and GFP expression in SINV-GFP-infected cells shown in B. Gamma-Tubulin was used as loading control. (TIF) [file ppat.1009549.s001.tif]

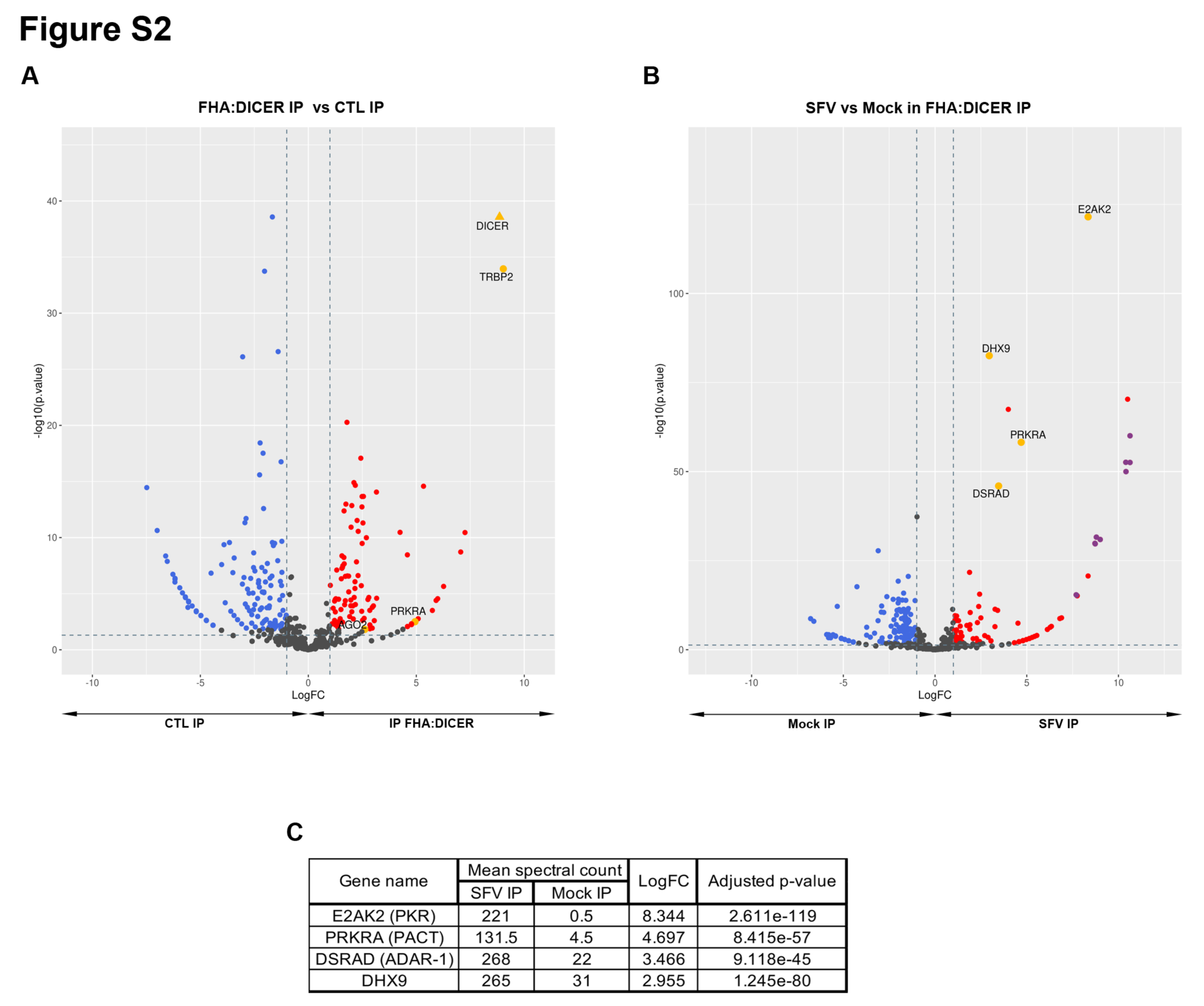

Supplement: S2 Fig — A. Volcano plot for differentially expressed proteins (DEPs) between HA IP and CTL IP in FHA:DICER mock-infected cells. Each protein is marked as a dot; proteins that are significantly up-regulated in HA IP are shown in red, up-regulated proteins in CTL IP are shown in blue, and non-significant proteins are in black. The horizontal line denotes a p-value of 0.05 and the vertical lines the Log2 fold change cutoff (-1 and 1). DICER and its cofactors (TRBP, PACT, AGO2) are highlighted in yellow. B. Left panel: Volcano plot for DEPs between SFV (MOI of 2, 6 hpi) and mock fractions of HA IP in FHA:DICER cells. Same colour code and thresholds as in A were applied. Proteins that are discussed in the text are highlighted in yellow and SFV proteins in purple. C. Summary of the differential expression analysis of SFV vs mock fractions from HA IP in FHA:DICER cells. The analysis has been performed using a generalized linear model of a negative-binomial distribution and p-values were corrected for multiple testing using the Benjamini-Hochberg method. (TIF) [file ppat.1009549.s002.tif]

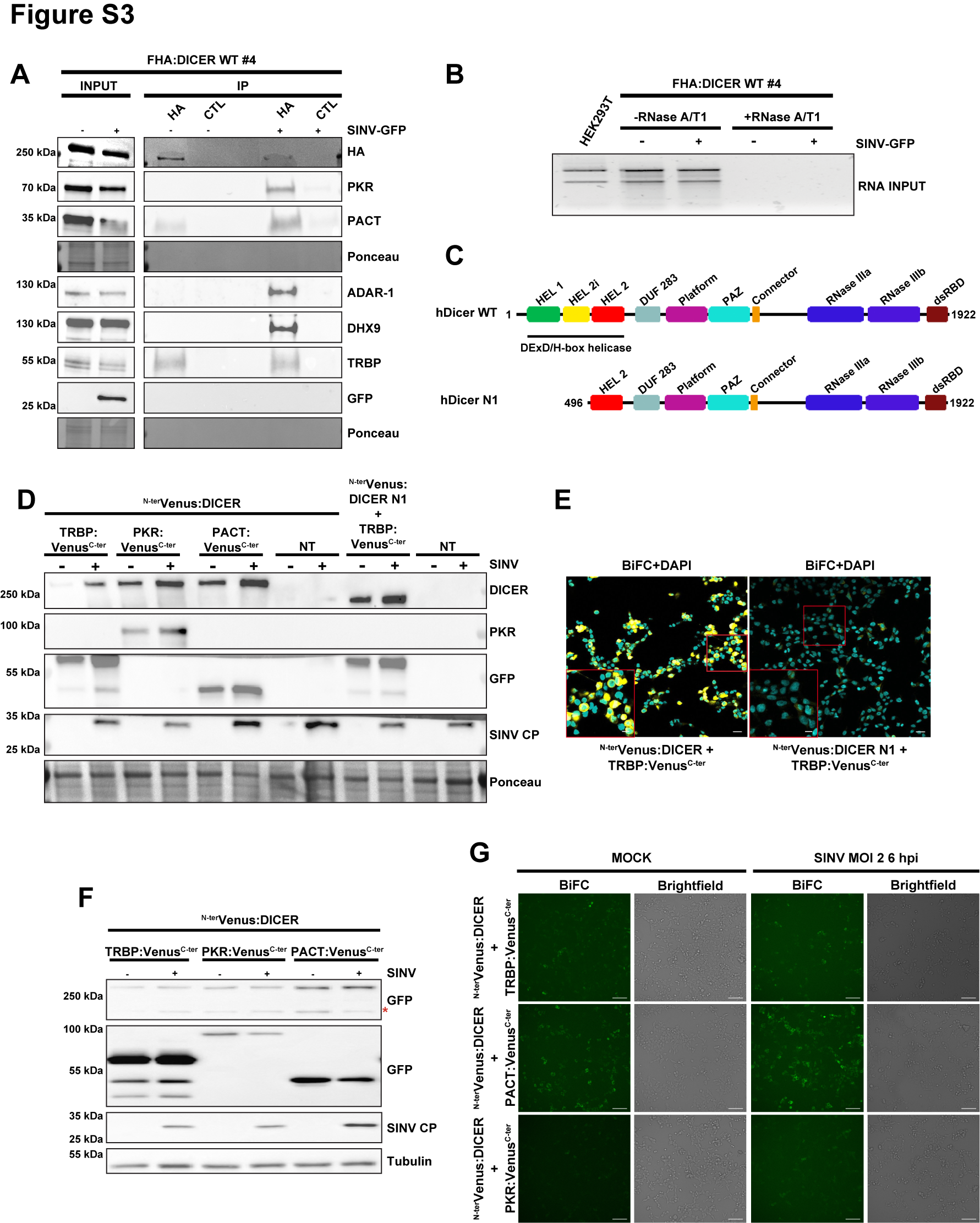

Supplement: S3 Fig — A. FHA:DICER WT #4 cells were infected with SINV-GFP at an MOI of 0.02 for 24 h and a HA co-IP was performed. Eluted proteins were resolved by western blot and IP efficiency was assessed using an HA antibody. In parallel, co-IPed proteins were visualized using appropriate antibodies. GFP antibody was used to verify the infection and Ponceau staining serves as loading control. B. 1% agarose gel analysis of RNA extracted from INPUT of the co-IP in Fig 3B. Ribosomal RNA integrity was compared to a control HEK293T cell line. RNAs were revealed using ethidium bromide under UV. C. Schematic representation of Human DICER proteins used for BiFC positive and negative controls. The different conserved domains are shown in colored boxes. DUF283: Domain of Unknown Function; PAZ: PIWI ARGONAUTE ZWILLE domain; dsRBD: dsRNA-binding domain. hDICER WT is the full-length protein. hDICER N1 is deleted of the first N-terminal 495 amino acids. D. Expression of BiFC plasmids was assessed by western blot. DICER proteins (WT and N1) and PKR were visualized using antibodies targeting endogenous proteins, whereas TRBP and PACT were detected using GFP antibody. Antibody targeting the SINV coat protein (CP) was used as infection control. Ponceau staining was used as loading control. E. Positive and negative BiFC controls on fixed NoDiceΔPKR cells. After co-transfection, cells were infected with SINV at an MOI of 2 for 6 h and fixed. After fixation, cells were stained with DAPI and observed under confocal microscope. Merge pictures of BiFC and DAPI signals of SINV-infected cells are shown. A higher magnification of images showing the interaction represented by a red square is shown in the bottom left corner. Scale bars: 20 μm and 10 μm. F. Expression of BiFC plasmids was assessed by western blot. DICER, PKR, TRBP and PACT were detected using GFP antibody. Antibody targeting the SINV coat protein (CP) was used as infection control. Gamma-Tubulin was used as loading control. The asterisk correspo [file ppat.1009549.s003.tif]

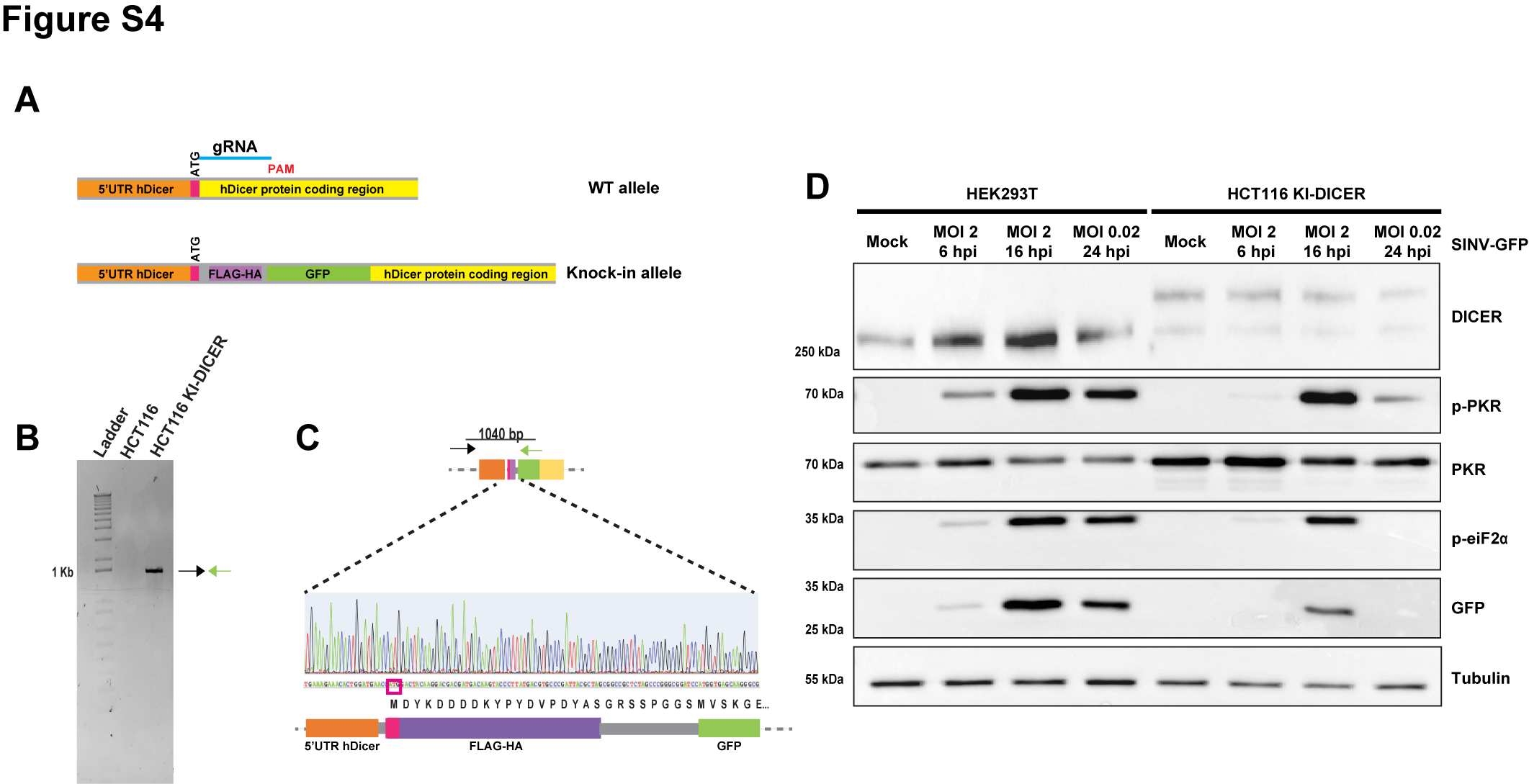

Supplement: S4 Fig — A. Schematic representation of DICER WT and Flag-HA(FHA)-GFP knocked-in (KI) alleles. FHA sequence is in purple, GFP in green, DICER 5’UTR in orange and DICER coding region in yellow. The gRNA used to generate the KI was designed to target the first coding exon of DICER gene. B. PCR on genomic DNA extracted from WT and KI cells. C. An oligo outside the homologous recombination region and an oligo within the GFP tag were used to verify the presence of a 1040 bp amplicon in HCT116 KI-DICER clone. Sequencing results corresponding to this region are shown. D. Western blot analysis of DICER, p-PKR, PKR and p-eIF2α expression in mock or SINVGFP-infected HEK293T and HCT116 KI-DICER cell lines at an MOI of 2 for 6 h or 16 h and 0.02 for 24 h. GFP antibody was used to verify the infection. Ponceau and gamma-Tubulin were used as loading controls. (TIF) [file ppat.1009549.s004.tif]

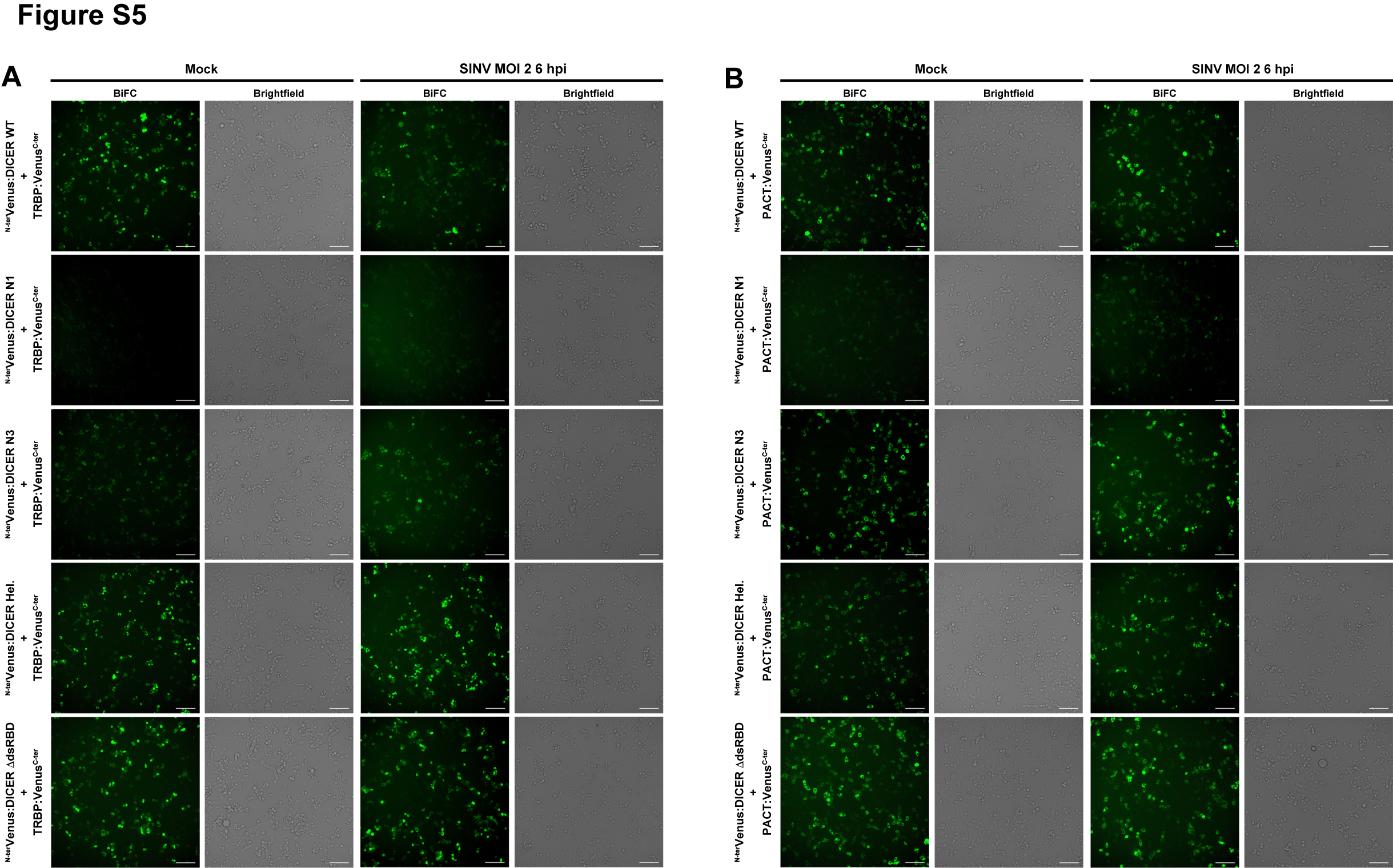

Supplement: S5 Fig — NoDiceΔPKR cells were co-transfected for 24 h with plasmids expressing the different versions of DICER proteins fused to the N-terminal part of Venus and either TRBP:VenusC-ter (A) or PACT:VenusC-ter (B). Cells were then infected with SINV at an MOI of 2 for 6 h and Venus signal was observed under epifluorescence microscope. The left panel corresponds to Venus signal and the right panel to the corresponding brightfield picture. Pictures were taken with a 5x magnification. hpi: hours post-infection. Scale bar: 100 μm. (TIF) [file ppat.1009549.s005.tif]

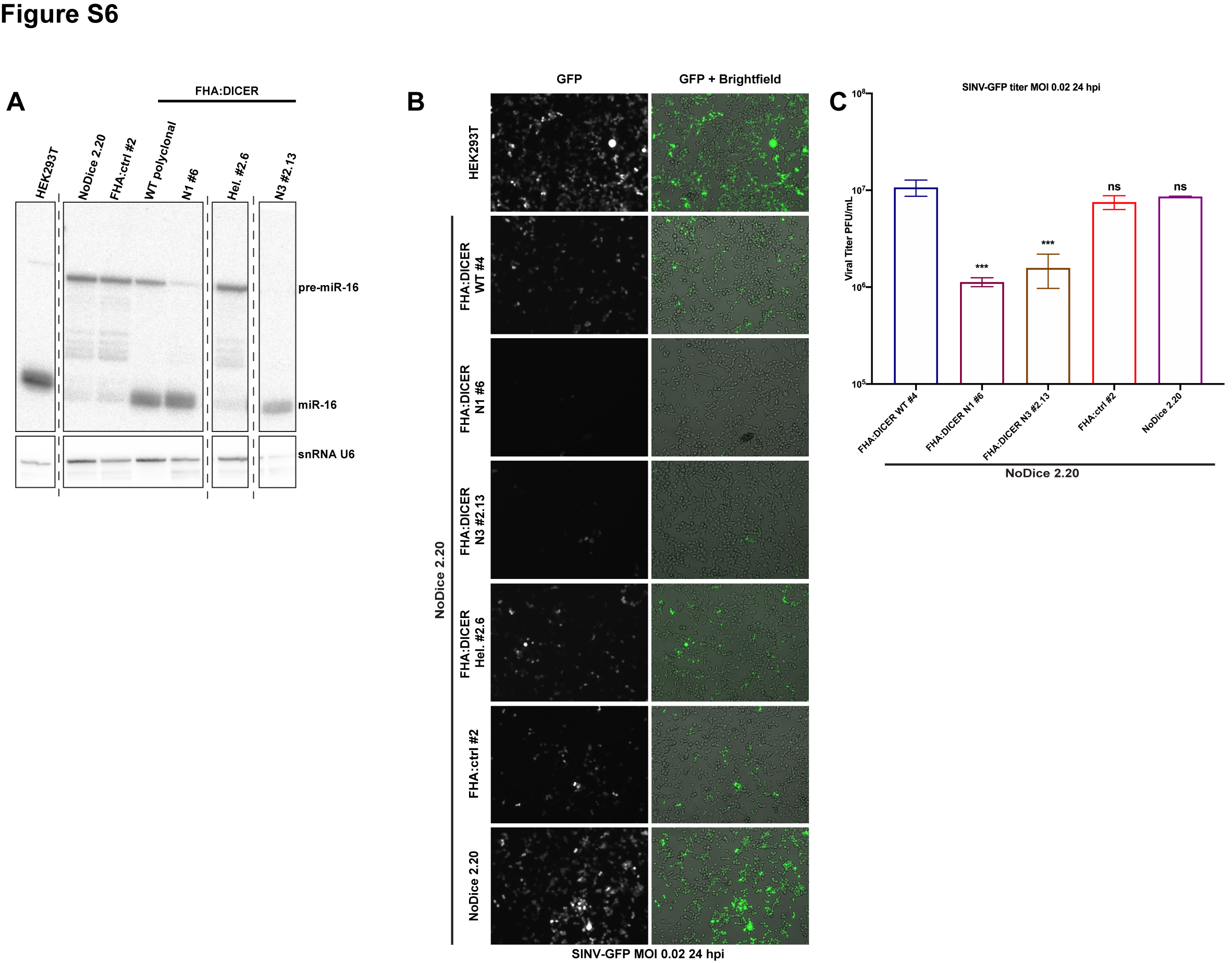

Supplement: S6 Fig — A. Northern blot analysis of miR-16 expression in HEK293T, NoDice 2.20, NoDice FHA:ctrl #2, FHA:DICER WT polyclonal, FHA:DICER N1 #6, FHA:DICER Hel. #2.6, and FHA:DICER N3 #2.13. Expression of snRNA U6 was used as loading control. B. Representative GFP fluorescent microscopy images of HEK293T, NoDice 2.20, FHA:DICER mutants cell lines infected with SINV-GFP at an MOI of 0.02 for 24 h. The left panel corresponds to GFP signal and the right panel to a merge picture of GFP signal and brightfield. Pictures were taken with a 5x magnification. hpi: hours post-infection. C. Mean (+/- SEM) of SINV-GFP viral titers over FHA:DICER WT #4 cells in FHA:DICER N1 #6, FHA:DICER N3 #2.13, NoDice FHA:ctrl #2 and NoDice 2.20 cell lines infected at an MOI of 0.02 for 24 h (n = 3) from plaque assay quantification. *** p < 0.001, ns: non-significant, ordinary one-way ANOVA test with Bonferroni correction. (TIF) [file ppat.1009549.s006.tif]
