# Supplementary material for: Human DICER helicase domain recruits PKR and modulates its antiviral activity
Source: PLoS Pathog. 2021 May 13;17(5):e1009549. doi: 10.1371/journal.ppat.1009549 (PMC8118307; doi:10.1371/journal.ppat.1009549)
Supplement: Attachment — Submitted filename: montavon_rebuttal_letter.pdf [file ppat.1009549.s014.pdf]

Replies to reviewers' comments

We would like to thank the editor and reviewers for their helpful comments and suggestions on our manuscript. We have carefully read them and addressed all of them in this revised version. Below are our replies to their comments. All modifications made in the text can also be seen in the document where track changes are highlighted.

<u>Major Issues: Key Experiments Required for Acceptance</u>

Reviewer #1: (No Response)

Reviewer #2: 1. The authors infected GFP-fusion SINV into HEK293T cells or Dicer knock-out cells and detected the fluorescence for the detection of viral replication. The human genome encodes single Dicer, so I am wondering whether the knock-out of Dicer influences the speed of cell growth. Does the difference of cell growth between WT and Dicer knock-out cells influence the viral replication? Is the decrease of fluorescence observed in Dicer knock-out cells (Fig.1A) a direct effect by Dicer for the regulation of viral replication?

> <u>*Reply:*</u> *the reviewer is right that Dicer knock-out has an effect on cell growth, however we do not believe that this directly influences viral replication as we show in Fig. 1C that the differences observed by microscopy are not confirmed when we measure the viral titer by plaque assay, which is quantitative. In addition, in another clone of the NoDice cells, the 4.25, we did not confirm this decrease of GFP fluorescence (Fig. S1B).*

2. The major effort of this manuscript seems to perform and describe clearly the results of immunoprecipitation and the BiFC approach. However, the most intriguing result with a lot of information is the proteomic analysis on Fig.2. The authors focused on PKR, PACT, ADAR1, and DHX9 in the subsequent analysis, but is it possible to look into the proteomic results more, not only these proteins but also others. My suggestion does not mean the requirement of additional experiments but might need additional informatical analysis such as GO analysis or Venn plot.

> <u>*Reply:*</u> *we thank the reviewer for this useful suggestion, we have now added some functional protein network analysis using the STRING database. This allowed*

us to visualize the known interactions between proteins identified in the mass spectrometry analysis, and to indicate the proteins involved in RNA metabolic processes, regulation of RNA metabolic processes and dsRNA binding. This new piece of information has been inserted in Fig. 2D and is discussed in the results section on page 7 lines 208-215 (in the manuscript with track changes highlighted).

3. Through the manuscript, there are several contradictions. For example, Dicer-PACT/Dicer-PKR interaction (Fig.3A vs Fig.3D), Dicer-TRBP interaction (Fig.3A vs Fig.4D), and GFP fluorescence level of FHA:ctrl #1 and FHA:Dicer WT#4 (Fig.1A vs Fig.6E) are inconsistent.

<u>Reply:</u> *it is true that it appears there could be some discrepancies between experiments, but we believe these can be explained as followed:*
i) regarding the difference between co-IP and BiFC experiments in Fig. 3A and 3D, we think that this is mainly due to the fact that in the BiFC experiments, both fusion proteins (DICER and PKR, or DICER and PACT) are transiently overexpressed, whereas in the co-IP experiments, we look at the endogenous proteins. This was mentioned for the DICER-PKR interaction on page 9, lines 285-288, and we added a mention regarding the DICER-PACT interaction on the same page, lines 288-289 (in the manuscript with track changes highlighted).
ii) the difference in DICER-TRBP interaction that the reviewer mentioned in Fig. 3A vs. Fig. 4D is less obvious, but we agree that it seems there is less signal for TRBP in the SINV-GFP HA IP lane. If we look at the signal in the HA western blot, it appears that the signal is also lower in the SINV-GFP + lane compared to the – lane. Therefore, we think that the difference in TRBP signal is only due to a lower efficiency of the IP in this condition.
iii) regarding the difference in GFP-fluorescence levels between FHA:ctrl #1 and FHA-DICER WT#4 in Fig. 1A and 6E, we regret that we do not see what the reviewer refers to since there is no FHA:ctrl #1 picture in Fig. 6C (not E). There is indeed a difference if one compares Fig. 1A and Fig. S6B regarding the difference in GFP fluorescence between the WT and the FHA:ctrl, but in the end what we can really refer to, and which is quantitative, is the measurements of viral titers. In that case, there is no significant difference between cells

expressing FHA:DICER WT or FHA:ctrl constructs be it in Fig. 1C, or in Fig. 6E.

4. In line 366, the authors mentioned that the deletion of dsRNA-binding domain did not influence the interaction of Dicer-PACT or Dicer-PKR, but RNase treatment decreased their interaction in Fig. 3B. Is dsRNA necessary for their interaction, or not?

<u>*Reply:*</u> *it is true that the deletion of the dsRBD domain in DICER does not influence its interaction with PACT or PKR. However, this domain has not been involved in the interaction of DICER with dsRNA. In fact, structural data indicate that this interaction is mediated by the PAZ domain (see MacRae et al. Science. 2006 Jan 13;311(5758):195-8 doi: 10.1126/science.1121638) or the helicase domain for Drosophila Dicer-2 (see Sina et al. Science 2018 Jan 19;359(6373):329-334 doi: 10.1126/science.aaq0921). We therefore believe that our two observations are not contradictory. Actually, we do mention in the text that this domain is more likely involved in protein-protein interaction than in interaction with dsRNA (see page 11 line 363 in the manuscript with track changes highlighted).*

5. The immunoprecipitation of endogenous PKR and Dicer/PACT (Fig.3C) might be changed to a clearer result.

<u>*Reply:*</u> *we agree with the reviewer that the blot showed in the earlier version of Fig. 3C was not optimal. We have now performed a new immunoprecipitation experiments and replaced panel C in Fig. 3 with a new one, which shows much more clearly that DICER can be IPed with the endogenous PKR only in SINV-infected cells.*

Reviewer #3: While the interaction between Dicer and PKR and its mapping to Dicer helicase domain is compelling, this study doesn't provide mechanistic insight into the role of Dicer-PKR interaction during viral infection. The authors demonstrated that the Dicer helicase domain was required for the interaction with PKR (Fig.5B, C and D). They then nicely showed that expression of Dicer helicase deletion mutants (Dicer N1 and Dicer N3) in NoDice HEK293T confer antiviral activity against SINV-GFP (Fig.6C, D and E). However, there is no experiments addressing the mechanism by which Dicer N1 and Dicer N3 exert their antiviral

activity and importantly whether it is dependent or not on PKR. This reviewer appreciates that a full dissection of the mechanisms at play is beyond the scope of this study. However, two experiments seem feasible to further investigate the mechanisms by which Dicer N1/N3 gained its antiviral activity.

• One possibility is that Dicer binds PKR through its helicase domain and thereby inhibits PKR antiviral activity. The expression of Dicer N1/N3, which are unable to bind PKR, would lead to an increased activation of PKR during viral infection. The authors should therefore test whether the expression of Dicer N1/N3 exhibits similar antiviral activities in the absence of PKR. This could be done by stably expressing these Dicer helicase deletion mutants in NoDice/PKR KO cells (used in all BiFC experiments) and test whether or not they still exhibit antiviral activity against SINV-GFP.

> Reply: *we thank the reviewer for this excellent suggestion, which we have followed in the revised version of this manuscript. We have generated NoDice/ΔPKR cells expressing FHA:DICER WT, N1 or N3 constructs and infected them with SINV-GFP. Very interestingly, our results show that the antiviral activity against SINV is completely lost in these conditions, thereby clearly indicating that PKR expression is essential in order to observe the antiviral phenotype of the helicase-truncated DICER mutants. These results have been added in a new Fig. 8 and are discussed in the results section (page 14-15 lines 459-478 in the manuscript with track changes highlighted) and in the discussion (page 17-18 lines 572-580 in the manuscript with track changes highlighted). Given the importance of this result, which clearly links the helicase domain of DICER and PKR, we have also changed the title of the manuscript to reflect this important finding.*

• Previous studies showed that the deletion of the helicase domain provides Dicer with an increased ability to process long dsRNA into siRNA and that expression of Dicer N1/N3 results in the accumulation of viral siRNA upon infection with Influenza virus (Kennedy et al., 2016; Tsai et al., 2018). The authors should therefore test whether the observed antiviral effect of Dicer N1/N3 on SINV-GFP is dependent on the RNAi pathway. An informative experiment would be to knock-down (or knock-out) Ago2 in NoDice FHA:Dicer N1 # 6 and NoDice FHA:Dicer N3 #2.13 (Fig. 6D and E) and test whether the antiviral activity of Dicer N1/N3 against SINV-GFP is impacted or not by the inactivation of the RNAi pathway.

Reply: again, thanks for this very pertinent comment. We have performed the suggested experiment, and the results clearly indicate that AGO2 is not implicated in the antiviral effect of either DICER-N1 or DICER-N3. Therefore, we can say that it does not seem that the observed phenotype is RNAi-dependent. These new results are shown in a new Fig. 7 and discussed in the results section (page 14 lines 436-457 in the manuscript with track changes highlighted) as well as in the discussion (page 17 lines 567-571 in the manuscript with track changes highlighted).

We believe that with the addition of these two new figures, and although we do not have a clear understanding of the exact mechanism involved, we can more confidently say that DICER modulates PKR antiviral activity by recruiting it via its helicase domain.

Minor Issues: Editorial and Data Presentation Modifications

Reviewer #1: This new version still sets up well the ongoing questions of whether RNAi is a relevant antiviral response in mammals, but exactly how the current work fits into this overall field was obscure. I believe the authors simply want to describe a novel interaction between RNAi and canonical mammalian antiviral response, and that Dicer could even be inhibitory in some situations. But, this could be made more clear in the Abstract/Discussion, and if I am missing some other points regarding this central question, perhaps these could also be stated directly in a more straightforward manner.

Reply: we thank the reviewer for this remark, and we agree that we did not explicitly write what our findings brought to the field. With the new experiments performed and described in Fig. 7 and 8 as well as in the text (see reply to Reviewer 2), we now have a better idea of how important the recruitment of PKR by DICER helicase domain is during SINV infection. We have therefore changed the title of our manuscript and made our point clearer in both the Abstract and in the Discussion.

Reviewer #2: (No Response)

Reviewer #3: • The authors nicely show that the interactions between Dicer and PKR, p-PKR, DHX9 and PACT are sensitive to RNAse A/T1 treatment demonstrating that these interactions are RNA-dependent. RNAse A/T1 specifically digest single-stranded RNAs, while PKR, ADAR1, TRBP, PACT are dsRNA-binding proteins. It would benefit the study to include an experiment in which the extracts are treated with a dsRNA-specific RNAse (RNase III) to test whether these interactions that are induced upon viral infection also depend on the accumulation of dsRNA generated during viral replication.

Reply: the salt concentration in the buffer used for the RNase treatment is 140 mM, it has been shown that at salt concentration below 300 mM, RNase A could cleave both single-stranded and double-stranded RNA. Therefore, our experimental conditions should remove both ss and dsRNA. While it is true that we do not formally show the strict dependency on dsRNA alone, we can thus at least partially reply to this concern. We added a sentence as well as references related to this point in the results section on page 8, lines 246-248 (in the manuscript with track changes highlighted).

• Page 7, line 146: the authors mention that "Dicer antiviral effect is not reproducible in an independent clone", but the data shows a Dicer proviral effect in some of the clones rather than an antiviral activity. The GFP signal from SINV-GFP infection is indeed reduced (and not increased) upon infection of NoDice cells 2.20 transduced or not with a lentiviral control.

Reply: this is a wording mistake, we wanted of course to write "DICER proviral effect". This has been corrected.

• Fig.S3D: The authors should show a larger portion of the immunoblot probed with anti-GFP antibody in order to also show the level of expression of PKR:Venus Cter.

Reply: this can be seen in the PKR immunoblot, since these are NoDicer/ΔPKR cells, the observed signal can only come from the PKR:Venus Cter fusion.

• Fig.S4D: the Ponceau staining is not informative in this case as it doesn't show much proteins in each well. As a loading control using anti-tubulin antibody was also performed and included in the figure, the authors should consider removing the Ponceau staining from this figure.

<u>Reply:</u> we agree with the reviewer that this panel is not informative, and we removed it.

• Figure 4D: the immunoblot showing the co-IP of Dicer with TRBP is not placed together with the co-IP of Dicer with PKR, PACT and DHX9 suggesting that these are 2 independent experiments. If it is the case, the level of Dicer (probed by an anti-HA antibody) in all fractions should also be included for this independent experiment. If these two sets of immunoblots are not independent experiments but are two membranes from the same experiment, this should be specified in the figure legend.

<u>Reply:</u> the TRBP immunoblot is from the same experiment but was done on a different membrane. We corrected the figure accordingly and indicated this in the figure legend.

• Figure 5B: there is a band visible in all wells of the "HA-IP" probed with anti-GFP antibody while all samples are not infected with SINV-GFP.

<u>Reply:</u> this is an aspecific band that migrates faster than GFP. For clarity reasons and to avoid any confusion, we removed the GFP immunoblot from Fig. 5B.

• Figure 5C: the molecular weight marker on the left-hand side of the immunoblot showing the input samples probed with anti-FLAG antibody might be incorrect as it shows that Dicer N3 runs at 70kDa. The "70kDa" label is likely part of the immunoblot just below and there is another "250 kDa" label under the "70kDa" that is likely a mistake and should be removed. The immunoblot of input samples probed with anti-FLAG antibody and showing the proteins in the range of 70kDa is not clear as it is difficult to see the expression of the "Dicer Hel." construct among the additional bands.

<u>Reply:</u> this is indeed a mistake; we corrected the figure accordingly. Regarding the problem with visualization of the DICER Hel. band in this panel, we agree it is difficult to see but there is a band that we indicated with a red asterisk in

the revised figure. In addition, the DICER Hel. band can be seen clearly in the HA IP, confirming the correct expression of this mutant isoform.

• Typo: Page 3, line 53: interferon-stimulated genes.

<u>Reply:</u> *typo corrected.*
