# Supplementary material for: Human DICER helicase domain recruits PKR and modulates its antiviral activity
Source: PLoS Pathog. 2021 May 13;17(5):e1009549. doi: 10.1371/journal.ppat.1009549 (PMC8118307; doi:10.1371/journal.ppat.1009549)
Supplement: Attachment — Submitted filename: montavon_response_to_reviewers.docx [file ppat.1009549.s013.docx]

**Replies to eLife reviewers’ comments.**

*We would like to thank the editors and reviewers for their helpful comments and suggestions on our manuscript. We have carefully read them and addressed most of them in this new submission, below are our replies to their comments. All modifications made in the text can also be seen in the document where track changes are highlighted.*

**Reviewer #1** (General assessment and major comments (Required)):

In this manuscript, Montavon et al investigate the Dicer interactome during Sindbis virus (SINV) infection. They identify a number of protein factors that specifically bind to Dicer in SINV-infected HEK293T cells and focus their investigation on PKR. Using both IP and BiFC approaches they confirm this interaction and show that it requires the Dicer helicase domain. They also show that SINV replication is inhibited in Dicer knockout cells, in contrast to previous publications. This is a well written manuscript describing carefully performed experiments with appropriate controls and makes an important contribution to the literature. However, several points need to be addressed.

*Reply: We would like to thank the reviewer for her/his positive comments, we address the specific points below.*

Major comments

1. The authors refer to the difference between their data and several earlier studies showing that viral replication in somatic mammalian cells is not affected by Dicer knockout. Some discussion of possible explanations for these discrepancies would be useful, in particular because Bogerd et al (J.Vir 2014) show no effect on a SINV infection timecourse using the same NoDice cell line. Given the different observations, this study would also benefit from confirmation of this result in different clones of the NoDice and FHA-Dicer cell lines.

*Reply: We agree with the reviewer that it was surprising to observe a difference of virus replication in the absence of Dicer, especially when this was not observed by others. This was indeed a critical point that we thoroughly addressed in our revision. In order to make sure that the effect we observed was not an artifact, we generated another FHA:ctrl cell line in the NoDice 2.20 background. Both FHA:ctrl clones showed a decrease of SINV infection compared to HEK293T cells (Fig. S1B, C). However, we also studied another clone of the NoDice cells, namely the NoDice 4.25 cell line, and our results indicate that in this clone the absence of Dicer does not result in a drop of SINV accumulation. Therefore, even if there is a tendency to a decrease of SINV infection in the absence of Dicer, it does not appear to be robust.*

*We have also tested other clones of the FHA:DICER cell line, namely clone #17 and a polyclonal line to make sure that they behaved similarly to HEK293T cells (Fig. 1A, C, D) and that the results obtained in the clone FHA:DICER #4 were representative.*

*Taken these new results into account, we have re-written this part of the manuscript to say that the tagged version of Dicer we are using is functional and can be used to study its interactome in the context of SINV infection, but we no longer refer to the antiviral effect of the lack of Dicer in this cellular context (see Fig. 1, S1 and text on lines 118-156).*

2. This manuscript shows some conflicting observations on whether binding of PKR and PACT to Dicer is affected by SINV infection. The IP experiments show that both proteins only interact in SINV-infected and not mock-infected cells (fig 3A, S3A), whereas in the BiFC experiments PACT-Dicer interaction is unaffected by viral infection (fig 3C). Moreover, PKR interaction with Dicer as seen by BiFC is increased by SINV infection in some experiments (fig 3C) but not in others (fig 4C, Dicer WT). The BiFC experiments are all carried out in the absence of endogenous Dicer and PKR. It is possible that this could affect the observed interactions between transfected Dicer and TRBP/PACT, particularly if TRBP, PACT and PKR all interact with the same region of Dicer. This needs some discussion, and ideally a repeat of the TRBP/PACT experiments in the background of endogenous PKR expression. It would also be helpful to repeat at least some of the IP experiment from figure 4B in mock-infected conditions for comparison.

*Reply: It is true that there appears to be some discrepancy between the co-IP experiments and the BiFC experiments. We believe that this is mainly due to the fact that in the later, we transiently overexpress the proteins in fusion with fragments of the Venus protein. This can explain why we detect some interaction of Dicer and PACT in Mock-infected cells by BiFC, as well as the differences observed between BiFC experiments (Fig. 3D and 5D). Ideally, we would have to stably express the Venus fusion proteins to be in the same context as the co-IP experiments, but this is not possible since this could result in an increase of aspecific signal due to the self-interaction of Venus-Ct and Nt parts, which can happen when the two fragments are co-expressed for extended time periods. This being said, even if it sometimes appears that there is slightly more fluorescent signal for the Dicer-PKR BiFC in infected cells, it is difficult to quantify, and we therefore do not make any claim along that line.*

*Regarding the fact that all BiFC experiments are carried out in the absence of Dicer and PKR, we agree that this could indeed impact the interactions between Dicer and TRBP or PACT. We have therefore repeated the BiFC experiment in HEK293T cells, where both Dicer and PKR are expressed, and we obtained the same results as in the NoDICE/deltaPKR cells (see Fig. S3G and text lines 267-270).*

*Finally, we have now done the co-IP experiments in mock-infected conditions to compare the results with the ones obtained in SINV-infected conditions. The results can be seen in Fig. 5B and indicate that in this context, Dicer WT only weakly interact with PACT and PKR (discussed in the text lines 328-331).*

3. While I appreciate that a full dissection of the mechanism by which Dicer-PKR interaction affects viral infection is beyond the scope of this study, it would benefit from some analysis of whether Dicer affects PKR function during SINV infection. It would be straightforward to determine whether phosphorylation of PKR and a relevant substrate such as eIF2alpha following SINV infection is changed in the NoDice cell line.

*Reply: We agree with the reviewer that it would be good to show that Dicer indeed affect PKR function during SINV infection. However, these are experiments that are difficult to put in place because based on our data it seems that PKR phosphorylation itself is not affected by the expression of a helicase-truncated Dicer (Fig. 5C input). However, we also show that these helicase mutants do not interact with p-PKR (Fig. 5C HA IP). It could be therefore that PKR is still phosphorylated upon SINV infection, when the virus dose is high enough, but might be sequestrated by Dicer WT, but not by the N1 or N3 mutants, and prevented from acting efficiently upon its substrates (see text lines 448-455). The exact nature of this substrate would have to be determined since it might not necessarily be eIF2a, and this would require much more work to sort out. In addition, there is also the possibility that the phenotype observed in cells expressing Dicer N1 or N3 is independent of PKR and is directly linked to Dicer processivity, and this is a hypothesis that we now also mentioned in the discussion (see text lines 487-493).*

*It seems therefore that it will require more work to fully decipher how the helicase domain of Dicer participates in the response to SINV infection and this would be outside of the scope of this manuscript.*

Reviewer #1 (Minor Comments):

Minor comments

1. The identity of the negative control cell line is not entirely clear. It is referred to as NoDice but this is also given as the name of the parental Dicer KO cell line obtained from the Cullen lab. However, the text implies that the negative control used in the experiments was obtained by lentiviral transduction of the NoDice cells with an empty vector. Could this be clarified, for example by using a different nomenclature?

*Reply: We apologize for the confusion. This part has now been modified extensively (see reply to the first comment) and we have clearly labelled the various cell lines used throughout the study. Thus, the negative controls that have been generated by transduction of an empty vector are now referred to as FHA:ctrl.*

2. The GFP+brightfield image for NoDice cells in figure S1B is very overexposed.

*Reply; This picture has been removed from the revised version of the manuscript as we have performed more experiments to characterize the phenotype of the NoDice cells (see reply above).*

**Reviewer #2** (General assessment and major comments (Required)):

In this manuscript, Montavon and colleagues present evidence that Dicer has a pro-viral activity in the HEK293T background and using a sophisticated well-powered mass spec proteomic approach, that Dicer interaction partners are dynamic and it interacts with a wide variety of interaction partners differentially upon infection. They especially pursue PKR and using different experimental techniques confirm that PKR differentially stably associates with Dicer upon viral infection. The authors further show that a mutant of Dicer that is able to restore miRNA biogenesis in the NoDice cells, fails to interact with PKR or rescue the pro-viral activity of Dicer. Overall, the findings of this work are interesting and contribute to an ongoing area of virology regarding the contribution of RNAi machineries to the vertebrate antiviral defense response.

Strengths of this manuscript

• The experiments are generally well-performed using state-of-the-art technologies

• The conclusions are not overstated and appropriate for the data provided

• This contributes interesting new insight of yet another interaction between the RNAi machinery and classic mammalian antiviral defenses

*Reply: We would like to thank the reviewer for his nice and positive comments about our work.*

Critiques

1. Major experiments are performed in the single parental cell type of HEK293T. This is necessary since there are few other options for parental cell types that can tolerate Dicer knockout, but nonetheless, this opens up the possibility that what is observed may not apply widely to other mammalian cells types.

*Reply: We agree with the reviewer that this was a major limitation of this work. To address this point, we have now added new results indicating that the interaction between Dicer and PKR, PACT or DHX9 during SINV infection is not restricted to HEK293T cells. We used the colorectal cancer cell line HCT116, which has been shown to be highly amenable to gene editing (Golden et al. Nature 2017, doi 10.1038/nature21025), and we tagged the endogenous Dicer protein by CRISPR-Cas9 mediated recombination with a FLAG-HA GFP at its Nter extremity. This cell line, which we named HCT116 KI-DICER, was then infected or not with SINV at a MOI of 0.1 for 24h, and Dicer was immunoprecipitated using an anti-HA antibody. Our results indicate that in HCT116 cells also, the interaction between Dicer and PKR, PACT or DHX9 is only present in infected cells. We can therefore be confident that the Dicer-PKR interaction, which is the key result of our manuscript, can be observed in cells other than HEK293T, and more importantly, also happens with the endogenous Dicer. These new results are shown in Fig. 4 and S4 and discussed in the text lines 282-315.*

2. The mechanism for how Dicer is pro-viral in these cells is only partially devised from the studies presented here. Although it is interesting that the N1 mutant Dicer both lacks the ability to associate with PKR and also loses its pro-viral activity, it is unclear if these two observations are linked, or whether alternative activities of the N portion of Dicer are responsible.

*Reply: Yes, it is perfectly possible that there are alternative activities of the helicase domain of Dicer and that the observed pro-viral activity is not solely mediated by its interaction with PKR. In fact, we also show that Dicer interaction with ADAR1 is increased during SINV infection, so it could be that there are several cofactors involved, and it might take some time to fully dissect the exact mechanism. We also show that expression of the helicase domain alone confers a phenotype similar to cells expressing the full-length Dicer (Fig. 6). While this does not answer the question of the mechanism, it does reinforce the notion that this portion of the Dicer protein might be involved in functions other than the canonical ones usually attributed to this protein.*

3. The antiviral nature of NoDice cells versus the rescued FHA-Dicer appear to have substantially different levels of rescue depending on the experiment. Figure 1E shows an impressive approximately 50-fold effect, while Fig. 5E shows only a 3-fold effect. Although there might be variations in the exact experiments to explain this, this seems like a wide variation in magnitude that affects how important to weight the overall observations of Dicer's newly-discovered pro-viral activity presented here.

*Reply: We agree that there were variations between experiments, and we have now carefully addressed this in the revised version of our manuscript. As indicated in our reply to Reviewer 1’s first comment, we have now tested other NoDice clones and our results indicate that the pro-viral activity of Dicer does not appear to be a robust phenotype (which could explain the variations we had). However, the antiviral activity of a helicase deleted Dicer is robust and can be observed with two independent constructs (N1 and N3). We have therefore reformulated the message of our manuscript accordingly and removed our statement regarding the pro-viral effect of Dicer.*

Reviewer #2 (Minor Comments):

4. Figure 3C, I believe the authors claim the bottom panels show increased fluorescence upon SINV infection, but this is difficult to see to my eye. If this is what they intend to claim, can they provide quantification for this? Related to this, in Fig. 4C, why is the BIFC fluorescent signal strong in the top panels whether mock or infected. Should this not be increased in the infected cells where PKR should show increased association with Dicer?

*Reply: Indeed, in some experiments, we noticed an increase in fluorescence for the Dicer-PKR interaction upon SINV infection. However, and as indicated in our reply to Reviewer 1 Comment 2, these experiments are performed by transient overexpression of the Venus fusion proteins, which could translate in variations between experiments, and also allow the observation of interactions that is not seen when the proteins are expressed at physiological levels. This likely explains why for example in Fig. 4C, we see a strong signal due to the Dicer-PKR interaction in the mock-infected cells. We have removed from the text our claims that we could observe an increase in fluorescence in BiFC experiments for Dicer-PKR in infected cells.*

5. All the primary data of MS that is provided in Excel files seem to be capped at top 100 hits. However, many of the results report more than 100 hits that are relevant (e.g., 258 hits differentially IP'd in Figure 2A but only 100 hits provided in Table S1?). Please provide all hits.

*Reply: We deliberately decided to show only the top 100 hits in the provided supplementary tables because all the mass spectrometry data has been uploaded to the ProteomeXchange Consortium via the PRIDE partner repository. It can be accessed with the dataset identifier PXD019093 and 10.6019/PXD019093 and the reviewer can login using the following identifiers if needed:*

*Url: https://www.ebi.ac.uk/pride/login*

*Username: reviewer13954@ebi.ac.uk*

*Password: XDPZgUvx*

*Once the manuscript is published these data will of course be available to the public.*

**Reviewer #3** (General assessment and major comments (Required)):

The paper by Pfeffer and colleagues describes a possible interaction between the helicase domain of Dicer and PKR, which has implications on the role of RNAi in viral infection.

The study is interesting and intriguing. However, since the paper really centers only around the interaction between the proteins, there are two aspects that should be included. One is to show a direct interaction in vitro. These proteins were expressed recombinantly previously, so should be doable. Even the appropriate truncations would suffice. In addition, although the authors make a number of arguments that this interaction is likely not mediated by RNA. However, since the interaction is the crux of the paper, this should be shown for this case rather than conjecture.

*Reply: We would like to thank the reviewer for her/his positive comment and suggestions. Regarding the in vitro analysis of the interaction between Dicer and PKR, this is an interesting suggestion. However, we are not sure whether it would add more to the validation of this interaction. Indeed, we show by two different approaches that the proteins interact ex vivo (by co-immunoprecipitation) and in vivo (using BiFC). We agree that it would be interesting to show that they can interact directly when expressed as recombinant proteins, but at the same time this would be artificial and would not necessarily reflect the situation in the cell.*

*We do agree however that it is important to show whether the interaction is mediated by RNA or not. In this revised version, we have performed experiments to answer to this point. The results indicate that, while the interaction appears to be mediated by RNA, a small fraction of the Dicer-PKR complex might be interacting in an RNA-independent manner (see Fig. 3B and text lines 218-231). We therefore hypothesize that RNA, probably of viral origin, brings Dicer and PKR together and facilitates their interaction.*
